# ‘Antimicrobial resistance is invisible. I am not.’

**DOI:** 10.2807/1560-7917.ES.2024.29.47.2400762

**Published:** 2024-11-21

**Authors:** Guido Werner, Muna Abu Sin

**Affiliations:** 1 Robert Koch Institute, Wernigerode, Germany; 2 Federal Ministry of Health, Berlin, Germany; 3 Robert Koch Institute, Berlin, Germany

Nearly 5 million deaths associated with bacterial antimicrobial resistance (AMR) in 2021, including more than 1 million deaths directly attributable to bacterial AMR [1], underlines that AMR is not a silent pandemic, but a global public health and development threat [2]. Each year on 18 November, the European Antibiotic Awareness Day takes place during the World Antimicrobial Resistance Awareness Week (WAAW), reminding us of the acute global crisis posed by AMR. The year 2024 has been marked by a United Nations High-Level Meeting on AMR and the adoption of a political declaration calling upon member states and the Quadripartite Organizations, namely the Food and Agriculture Organization of the United Nations (FAO), the United Nations Environment Programme (UNEP), the World Health Organization (WHO), and the World Organisation for Animal Health (WOAH), to strengthen their efforts and tackle AMR collaboratively within a One Health approach.

“Educate. Advocate. Act now.” is the theme of WHO’s 2024 WAAW campaign [3]. It highlights how important continuous education of and dialogue with policymakers is to keep AMR high on the political agenda, to raise awareness, and to translate commitments into tangible actions. To illustrate that AMR does not merely affect statistics, the recently launched campaign, ‘Antimicrobial resistance is invisible. I am not.’ of the WHO Task Force of AMR Survivors puts patients’ perspectives and stories at the centre [4]. It demonstrates how AMR impacts all of us – as patients and relatives who trusted in antibiotics that no longer work for them and healthcare professionals who face diminishing options for treatment. Antimicrobial resistance as well as sustainable and affordable access to antibiotics affects us all, and in low- and middle-income countries the lack of access to essential antibiotics exacerbates the already high burden of infectious diseases. This underlines the importance of preventing infections by vaccinations and infection prevention and control measures including safe water, sanitation and hygiene practices [5]. Also, medical advances we take for granted such as cancer treatment, surgery and organ transplantation cannot be ensured when we lose the effectiveness of antibiotics.

A series of articles in two consecutive issues of *Eurosurveillance* detail a broad range of research activities addressing AMR and adding to the body of evidence to guide and inform interventions to tackle the problems that we face. The topics include antibiotic consumption, AMR surveillance, current and future research priorities in this area, and genome-based and pathogen-centred approaches responding to the challenge of AMR within and across the human, animal and environmental sectors.

A study from 10 non-university hospitals in Germany by Först et al. [6], reveals concerning trends in prescription practices. Among the 8,560 patients evaluated, 33% received antimicrobials, primarily beta-lactam/beta-lactamase inhibitors and cefuroxime for prophylaxis. Adherence to quality indicators was inconsistent, with documentation and streamlining of therapy showing particularly low compliance. These findings underscore the need for strengthened antimicrobial stewardship (AMS) programmes in non-university hospitals, supported by clear frameworks to improve quality-driven prescribing of antimicrobials. In Belgium, Kelly and colleagues [7] investigated antibiotic consumption among persons aged 65 years and older in and outside nursing homes and found consumption to be consistently higher in institutionalised populations, despite an overall decline in use over the study period from 2016–2022. This discrepancy widened during the COVID-19 pandemic, highlighting vulnerabilities in institutional settings where antimicrobial treatments often exceed recommended durations and quantities. This study shows that nursing home residents face unique infection risks and contribute notably to current AMR trends.

Metrics for antibiotic consumption impact the analysis of AMR trends and the effectiveness of AMS programmes. An evaluation of data from 24 European Union/European Economic Area countries provided by Rubinić et al. [8] highlights how different hospital activity-based denominators (e.g. bed-days and discharges) can provide more consistent and reliable insights compared with population-based measures like defined daily doses (DDD) per 1,000 inhabitants per day. Shifting to these refined metrics can enhance the targeting of stewardship interventions and improve cross-national comparisons.

A consensus among experts on AMR research priorities emphasises the urgent need for pathogen-specific and microorganism-centred data to guide the development of antibacterial vaccines and monoclonal antibodies as presented by Hassoun-Kheir et al. [9]. Prioritised targets include third-generation cephalosporin-resistant *Klebsiella pneumoniae* and *Escherichia coli* for bloodstream and urinary tract infections, as well as meticillin-resistant *Staphylococcus aureus* for surgical site infections. This targeted approach also aims to refine AMR burden assessments and align preventive strategies with the most pressing threats.

Genomic surveillance uncovered sustained transmission of carbapenem-resistant *Providencia stuartii* carrying the *bla*_NDM-1_ gene in Romania, marking a serious public health threat. The identification of multi-hospital clusters across regions by Linkevicius et al. [10] indicates that AMR can persist undetected and spread across healthcare facilities. Isolates from Romanian hospitals demonstrated clonal relatedness to isolates from a recent Europe-wide study [11]. Linkevicius et al. advocate for comprehensive reporting and genomic analysis of all carbapenem-resistant pathogens, also across borders, to implement reliable infection prevention and control measures. A study by the European Antimicrobial Resistance Genes Surveillance Network [12] analysed the emergence of *Escherichia coli* ST131 carrying carbapenemase genes across several European countries. It shows that spread of AMR is not limited to healthcare facilities but takes place in the community and underlines the importance of integrated analysis of epidemiological and genomic data to better understand and control transmission risks.

Plasmid-mediated AMR transmission can occur across sectors and reveals the interconnected nature of AMR across humans, animals, and the environment. A comprehensive analysis of 3,745 *E. coli* isolates from various European countries by Kaspersen et al. [13] demonstrated widespread resistance to critical antimicrobials, driven by both vertical and horizontal gene transfer. IncI1 plasmids, in particular, were identified as significant vectors for resistance spread between livestock and humans. These findings stress the importance of controlling plasmid dissemination to curb AMR evolution.

The body of research findings points to an urgent need for an innovative research agenda and for robust surveillance strategies across healthcare and community settings to inform interventions. From optimising prescription practices and refining consumption metrics to focusing on high-risk pathogens and addressing interregional transmission, a coordinated and comprehensive strategy is crucial to mitigate the global AMR threat. Let’s not forget what we are aiming for with all our AMR activities: to reduce the burden for patients and their families, to obtain state-of-the-art medical treatment options, and to tackle the threat posed by potentially untreatable infections and AMR, for the present and future generations.

The European Antibiotic Awareness Day 2024 calls upon all stakeholders – patients, healthcare providers, public health professionals, and policymakers – to pay attention to patients’ stories and research that provides the evidence for impactful actions. Patients’ experiences give the human face to the AMR crisis. It is essential to understand the impact of AMR on individuals’ health and well-being and to prioritise sustainable access to effective antibiotics, while simultaneously promoting responsible and prudent antibiotic prescribing and use as essential steps to protect the population from AMR and prevent AMR from claiming even more lives.

## Note

Guido Werner is Head of the Division Nosocomial Pathogens and Antimicrobial Resistances and of the German National Reference for Staphylococci and Enterococci at the Robert Koch Institute.

Muna Abu Sin is the Antimicrobial Resistance Ambassador of the German Federal Ministry of Health.
